# Rational Design and Preparation of Functional Hydrogels for Skin Wound Healing

**DOI:** 10.3389/fchem.2021.839055

**Published:** 2022-01-24

**Authors:** Ruinan Hao, Zhuoyi Cui, Xindan Zhang, Ming Tian, Liqun Zhang, Feng Rao, Jiajia Xue

**Affiliations:** ^1^ Beijing Laboratory of Biomedical Materials, Beijing University of Chemical Technology, Beijing, China; ^2^ State Key Laboratory of Organic-Inorganic Composites, Beijing University of Chemical Technology, Beijing, China; ^3^ Trauma Center, Peking University People’s Hospital, Beijing, China; ^4^ Key Laboratory of Trauma and Neural Regeneration, Ministry of Education, National Trauma Medical Center, Peking University, Beijing, China

**Keywords:** skin wound healing, hydrogel, wound dressing, bioactive materials, tissue regeneration

## Abstract

Skin wound healing often contains a series of dynamic and complex physiological healing processes. It is a great clinical challenge to effectively treat the cutaneous wound and regenerate the damaged skin. Hydrogels have shown great promise for skin wound healing through the rational design and preparation to endow with specific functionalities. In the mini review, we firstly introduce the design and construction of various types of hydrogels based on their bonding chemistry during cross-linking. Then, we summarize the recent research progress on the functionalization of bioactive hydrogel dressings for skin wound healing, including anti-bacteria, anti-inflammatory, tissue proliferation and remodeling. In addition, we highlight the design strategies of responsive hydrogels to external physical stimuli. Ultimately, we provide perspectives on future directions and challenges of functional hydrogels for skin wound healing.

## Introduction

As the largest organ of the human body, skin is the most important natural barrier to protect human from external injuries (Gao et al., 2019). Due to various types of internal and external factors, such as mechanical injuries, burns, chronic skin trauma caused by diabetes and malignant tumors, human skin is extremely vulnerable to damage. At present, it is still a great clinical challenge to effectively treat the cutaneous wound and regenerate the damaged skin. Cutaneous wound healing is a dynamic and complex physiological process, which generally includes four overlapping but different periods: hemostasis, inflammation, proliferation, and remodeling (Chen et al., 2021). These four healing stages involve interactions among various types of cells and their products, bioactive factors, and extracellular matrices (ECMs) (Xiao et al., 2019; Zhang et al., 2019). However, due to the imbalance of the physiological environment under severe conditions, the wound healing process may stop at one of the above stages, resulting in impaired cell function at the wound site and the failure of normal healing. Therefore, an accurate wound management is of great significance to promote wound healing.

One of the promising treatment strategies for promoting wound healing is to provide an artificial matrix as the wound dressing. Simulating the wound healing microenvironment, many wound dressing or skin substitutes have been developed. Specifically, hydrogels have attracted much attention as wound adjuvants because of their high porosity, interconnected macro-porous network, large specific surface area, and appropriate degradation rate, as well as their capability of maintaining moist microenvironment and absorbing tissue exudates (Wang Z. et al., 2019; Asadi et al., 2021). In addition, functional hydrogels can be obtained through rational design and preparation. Among them, injectable *in-situ* moldable hydrogels are gaining attention, which can be directly applied to irregular wounds (Xuan et al., 2021). A plain hydrogel is often not effective enough in repairing skin wounds by only serving as a covering material. Therefore, it is essential to integrate the hydrogel matrix with typical types of drugs, bioactive factors, cells, and/or specific genes to promote wound repair and accelerate skin regeneration.

**FIGURE 1:**
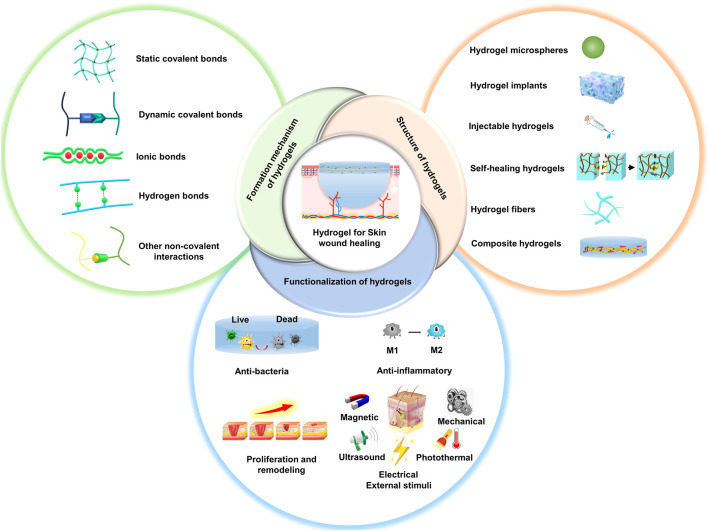
Schematic showing the rational design and preparation of functional hydrogels for skin wound healing.

The overview of this review is shown in Figure 1. We firstly introduce the design and construction of various types of hydrogels based on their bonding chemistry during cross-linking. Then, we summarize the recent research progress on the functionalization of bioactive hydrogel dressings for skin wound healing (Table 1). Finally, we provide suggestions and perspectives on future directions and improvement strategies for hydrogel wound dressings. We hope that this literature will provide ideas and references for research in the field of skin wound repair.

**TABLE 1:** Functional hydrogels for skin wound healing.

Hydrogels	Bond chemistry of hydrogels	Structure of hydrogels	Functionalization of hydrogels	Animal models	References
Gelatin/poly (vinyl alcohol) hydrogels	Dynamic phenylboronic acid−diol-ester bonds	Injectable hydrogel	Vancomycin-conjugated silver nanoclusters, pH-sensitive micelles loaded with Nimesulide	Diabetic rat model	Wang et al. (2021b)
Heparin-poloxamer hydrogels	Amido bond	Patch	Different growth factor (a-FGF and b-FGF)	Rat full-thickness skin defect model	Wu et al. (2016)
Bacterial cellulose/MXene hydrogels	Hydrogen bond, chemical covalent cross-linking bond, chain self-entanglement	Patch	Ti_3_C_2_Tx-MXene	Rat full-thickness skin defect model	Mao et al. (2020)
Calcium peroxide-GelMA hydrogels	Carbon-carbon covalent bonds	3D printed patch	Calcium peroxide	—	Erdem et al. (2020)
Bilayered thiolated alginate/polyethylene glycol diacrylate hydrogels	Disulfide bond, Carbon-carbon covalent bonds	Bilayered patch	The small extracellular vesicles (sEVs) secreted by bone marrow derived mesenchymal stem cells, and the sEVs secreted by miR-29b-3p-enriched bone marrow derived mesenchymal stem cells	Full-thickness skin defect model of rats and rabbit ears	Shen et al. (2021)
VEGF-decorated t-ZnO-laden hydrogels	Carbon-carbon covalent bonds	3D printed patch	Tetrapodal zinc oxide, VEGF	Full-thickness skin defect model	Siebert et al. (2021)
Poly (lactic-*co*-glycolic acid) microcapsules hydrogels	Imine crosslinking	Injectable hydrogel	TGF-β inhibitor	Rabbit ear and porcine skin wounding model	Zhang et al. (2021a)
Bio-multifunctional hydrogels	MgO-catechol, Schiff’s base bond	Injectable hydrogel	MgO	Full-thickness cutaneous defect and burn model	Tang et al. (2021)
Multifunctional double colorimetry-integrated polyacrylamide-quaternary ammonium chitosan-carbon quantum dots-phenol red hydrogels	Carbon-carbon covalent bonds, physical entanglement	Patch	Carbon quantum dots, phenol red	Rat skin defect model	Zheng et al. (2021)
The peptide modified nanofibers reinforced hydrogels	Schiff’s base bond	Composite hydrogel	Antimicrobial peptide (RRRFRADA)	Murine diabetic wound healing model	Qiu et al. (2021)
Sodium alginate-chitosan oligosaccharide-zinc oxide hydrogels	Schiff’s base bond	Patch	Zinc oxide nanoparticles	Second-degree scald wounds model	Zhang et al. (2021b)
Catechol/ε-polylysine hydrogels	Schiff’s base bond	Patch	Catechol, ε-polylysine	Burn Wound Infection Model	Xu et al. (2019b)
Polypeptide-based FHE hydrogels	Schiff’s base bond	Injectable and self-healing	stimuli-responsive adipose-derived mesenchymal stem cells exosomes	Diabetic rat model	Wang et al. (2019a)
Metallohydrogels	Coordination self-assembly	Patch	Amino acid, Ag^+^	Bacterial infection rat model	Song et al. (2020)
Hydrogel/polycaprolactone core/shell fiber scaffolds	Ionic bond	3D printed patch	Polydopamine, doxorubicin	Rat full-thickness skin defect model	Liu et al. (2021)
Gelatin-Tannic acid hydrogels	Hydrogen Bond	Patch	Tannic acid, allantion	Rat full-thickness skin defect model	Ahmadian et al. (2021)
Epigallocatechin-3-gallate-3-acrylamido phenylboronic acid complex-based polyacrylamide hydrogels	Boronate ester bond, phenylboronate ester	Patch	Epigallocatechin-3-gallate, 3-acrylamido phenylboronic acid	The diabetic rat model	Zhao et al. (2021b)
Injectable multifunctional hydrogels	Schiff’s base bond	Injectable hydrogel	ε-polylysine-coated MnO_2_ nanosheets, insulin	The diabetic rat model	Wang et al. (2020)
Polydopamine-modified graphene oxide hydrogels	Schiff’s base bond	Patch	Graphene oxide, ε-polylysine	The diabetic rat model	Tu et al. (2021)
Sprayable hydrogels	Carbon-carbon covalent bonds	sprayable	Cerium oxide nanoparticles, antimicrobial peptide	Infected rat model	Cheng et al. (2021)
Dual-dynamic-bond cross-linked antibacterial adhesive hydrogels	pH-sensitive coordinate bond, Schiff’s base bond	Patch	Protocatechualdehyde, ferric iron	Rat skin incision model	Liang et al. (2021)
Polyvinyl alcohol-iodine hydrogels	Hydrogen bond	Patch	Iodine	Rat skin incision model	Miao et al. (2021)
Cellulose-based adhesive hydrogels	Covalent bond, hydrogen bond, cation-π, π-π stacking, electrostatic interaction	Patch	—	Rat full-thickness skin defect model	Lu et al. (2021)

## Formation Mechanism of Hydrogels

### Static Covalent Bonds

Static covalent bonds of hydrogels are formed by chemical reaction or static cross-linking of complementary functional groups. Static covalent bonds are usually stable and robust, but cannot be recovered once broken. Though the self-healing ability of static covalent bonds is relatively weak, on the other hand, it can significantly improve the mechanical properties of hydrogels. For example, a double network hydrogel was prepared by oxidized methacrylic acid sodium alginate and collagen-polypeptide-functionalized carboxymethyl chitosan, which were based on the dynamic covalent Schiff’s base bond and UV irradiation to form a static covalent bond (He et al., 2021).

### Dynamic Covalent Bonds

Dynamic covalent bonds are usually reversible covalent bonds formed by dynamic crosslinking, including disulfide, imine, acyl hydrazone, phenylboronate ester, etc. (Yang et al., 2019). Compared with static covalent bonds, dynamic covalent bonds can be reversibly broken and reorganized under a certain external stimulus, such as pH, light, and heat (Chakma and Konkolewicz, 2019; Huang et al., 2020). Therefore, hydrogels with good shape recovery ability and excellent self-healing ability can be prepared by introducing multiple reversible bonds as cross-linking agents. For example, the catechol group in epigallocatechin-3-gallate can form two reversible borate ester bonds with phenylboronic acid, which can be used as a dynamic cross-linking agent to form hydrogel with acrylamide (Zhao X. et al., 2021). This dynamic reversible property endows the hydrogel excellent self-healing properties. Tang et al. (2021) used catechol-modified chitosan and oxidized dextran as the main chain segments of the hydrogels to form dynamic covalent bonds via the Schiff’s base reaction, while MgO and catechol-modified chitosan were chelated as another gel agent to form a double chemical bond hydrogel.

### Ionic Bonds

Electrostatic interactions can form ionic bonds, especially between metal ions and nonmetal ions. The formation of ionic bonds can enhance the properties of hydrogels to a certain extent, and different ions can also impart specific characteristics to hydrogels. For instance, magnesium ions bonded cross-linked hydrogels can effectively improve the attachment and enhance the bioactivity of primary osteoblasts *in vitro* (Yin et al., 2015). Hydrogels based on alginate and chondroitin sulfate are cross-linked by calcium ionic bonds (Fajardo et al., 2012). Under acidic conditions, the functional groups in both alginate and chondroitin sulfate are in neutral form, making the hydrogel network more hydrophobic and thus giving it a pH-dependent water absorption ability. The presence of iron ions can increase the strength of ionic bonds. For example, the carboxyl group in the copolymer of acrylamide and acrylic acid (PAMAAc) strongly interacts with iron through coordination interactions and then generates agar/PAMAAC-Fe^3+^ double network hydrogel (Chen et al., 2016). This highly coordinated interaction dramatically improves the self-healing ability and good fatigue resistance of the hydrogel.

### Hydrogen Bonds

Hydrogen bonding is the intermolecular force between hydrogen atoms and another atom with high electronegativity. Hydrogen bond complexes can be formed by the dense arrangement of hydrogen bonds between polymers. For example, the hydroxyl group of tannic acid acts as the hydrogen donor, and the carboxyl and amino groups of gelatin act as the hydrogen acceptor, forming abundant hydrogen bonds and generating hydrogels rapidly (Ahmadian et al., 2021). Although hydrogen bonds are unstable in an aqueous environment, they are stable under hydrophobic conditions. Hydrogels formed from methacrylic acid and methacrylamide can be cross-linked by hydrogen bonds (Wang Y. J. et al., 2019). Methyl groups are hydrophobic groups that cover hydrogen bonds and stabilize the hydrogel from attack by water molecules. Moreover, the dense and robust hydrogen bonding network gives the hydrogel super stiffness.

### Other Non-covalent Interactions

Other non-covalent bonds also contribute to the formation of hydrogels with a 3D network structure, including hydrophobic interaction (Liu X. et al., 2020), dipole-dipole interaction (Jia et al., 2017), π-π interaction (Li et al., 2010), electrostatic interaction (Zhang and Khademhosseini, 2017), and host-guest interaction (Sinawang et al., 2020), etc. The non-covalent bond is relatively weak, so multiple types of non-covalent bonds are often used at the same time to form a hydrogel. In addition, non-covalent bond can be applied as a complement to covalent bonds to form hydrogels. For example, an injectable micellar hydrogel was developed by combining Schiff’s base dynamic covalent bonds, hydrogen bonding, and π-π stacking interactions (Yang et al., 2020).

## Structure of Hydrogels for Wound Healing

### Hydrogel Microspheres

Compared to the traditional bulk hydrogels, hydrogel microspheres are smaller in size and can reach the nanoscale. Hydrogel microspheres can be used to deliver drugs, bioactive factors, and stem cells for tissue repair (Zhao Z. et al., 2021). The commonly used preparation methods of hydrogel microspheres are batch emulsion, microfluidic, lithography, electro jetting, and mechanical crushing (Daly et al., 2020). For example, Griffin et al. (2015) prepared polyethylene glycol-based hydrogel microspheres using a microfluidic emulsion.

### Hydrogel Implants

Hydrogel patches are the most common form of wound dressings. In addition to the use of molds to prepare wound dressing with regular shapes, 3D printing technology offers a new method for patch preparation. According to the shape of the wound, 3D printing can be used to prepare individualized patches conforming to the shape of the wound. Alizadehgiashi et al. (2021) used cellulose nanocrystals and methyl acrylamide-chitosan as printing inks to prepare hydrogel patches with homogeneous composition and mesh structure by 3D printing for active substance delivery, which is not possible with conventional patches for their incompetent in reaching the deeper layers of the skin. The bulged microstructure on the surface of the patch can penetrate well into the deep part of the damaged skin, realizing the profound delivery of bioactive factors to promote skin repair. Sun et al. (2021) prepared MXene-integrated microneedle array patches loaded with adenosine to achieve a controlled release under near-infrared (NIR) stimulation.

### Injectable Hydrogels

For irregular wounds, it is difficult for the traditional hydrogel patches to completely and perfectly cover and fit the shape and size of the wound (Gao et al., 2020). Injectable hydrogels offer a solution for the repair of a wound with irregular shapes. The fabrication of injectable hydrogels can be realized by regulating the bond chemistry of hydrogels. Dynamic covalent bonds, ionic bonds, hydrogen bonds, and other non-covalent bonds can provide ideas for the preparation of injectable hydrogels. For example, injectable hydrogels were fabricated through a Schiff’s base reaction between ε-polylysine-coated MnO_2_ nanosheets and insulin-loaded self-assembled aldehyde Pluronic F127 micelles (Wang et al., 2020).

### Self-Healing Hydrogels

Due to the low mechanical strength of hydrogel, it is easily damaged during application (Xuan et al., 2021). The development of self-healing ability is crucial for hydrogel patches. Similar to the preparation of injectable hydrogels, self-healing hydrogels can also be realized by reversible dynamic covalent bonds or non-covalent bonds. For instance, a self-healing hydrogel dressing was obtained through the formation of boronate ester (Zhao X. et al., 2021). When the two surfaces of the separated hydrogel contacted to each other, the rearrangement of the boronate ester bond could promote the self-healing of the separated hydrogel. Takashima et al. (2012) reported a self-healing hydrogel based on supramolecular host-guest interaction, which was prepared by copolymerization of acrylamide with the complex of β-cyclodextrin (host) and adamantane (guest) monomer. After being damaged, the hydrogel was self-healing through the host-guest interaction between β-cyclodextrin and adamantane. At room temperature, the mechanical properties of the self-healing hydrogel could be fully restored after contact at the cutting site for 24 h, showing excellent self-healing properties.

### Hydrogel Fibers

Compared with traditional hydrogels, hydrogel fibers have a 3D network structure with a higher specific surface area, which can simulate the structure and characteristics of the ECM and provide more sites for cell adhesion, migration, and proliferation (Li et al., 2021). The standard preparation methods of hydrogel fibers include electrospinning (Liu W. et al., 2020), 3D printing (Kong et al., 2020), microfluidic spinning (Cai et al., 2019), and dynamic polymer spinning (Chen et al., 2020). The most commonly used method is to prepare nanofibers by electrospinning and then cross-link the obtained fibrous membrane to improve its mechanical properties. Chen et al. (2019) successfully prepared gelatin methacryloyl (GelMA) fibers by electrospinning. They soaked the fibrous membrane in the solution containing initiator crosslinking to fabricate hydrogel fibers. Hydrogel fibers can be further functionalized by surface modifications to endow them with special functions. For example, Liu et al. (2021) used 3D printing technology to prepare core-shell hydrogel/Polycaprolactone fibers and grafted a layer of dopamine on the outermost layer to endow it with the ability of NIR-triggered drug release.

### Composite Hydrogels

Although hydrogels can absorb tissue exudates and maintain water balance at the wound site, most hydrogels have poor mechanical properties, limiting their biomedical applications (Teixeira et al., 2021). Qiu et al. (2021) incorporated the peptide modified fiber composite into the hydrogel, and the stability and mechanical strength of the hydrogel was greatly improved. In addition to poor mechanical stability, a plain hydrogel scaffold cannot mimic the complexity of the natural ECM, making the development of novel composite hydrogels critical. For example, gelatin nanofibers were combined with a photo-crosslinking composite hydrogel-loaded with epidermal growth factor (EGF) to prepare a bionic bilayer composite hydrogel scaffold (Zandi et al., 2021). In the scaffold, the nanofibers and the EGF-loaded composite hydrogel acted as the dermal and the epidermal layer, respectively.

## Functionalization of Hydrogels for Wound Healing

### Anti-Bacteria and Anti-inflammatory

When skin injuries occur, microorganisms can easily invade and cause severe wound infections, thus preventing wound healing. Some hydrogel materials have inherent antibacterial properties, such as chitosan (Chi et al., 2020) and modified chitosan (Lu et al., 2020). However, these materials alone are less effective as antimicrobial agents, and therefore the addition of antimicrobial active ingredients is required to achieve an excellent antimicrobial efficacy. The most common solution is to load antimicrobial agents, antibiotics, and/or metal nanomaterials into the hydrogel to treat the infection at the wound site. Wang et al. (2021b) reported an antibacterial hydrogel prepared by coupling vancomycin with silver nanoparticles. ZnO nanoparticles were also loaded in a hydrogel to endow it with the antibacterial ability (Zhang M. et al., 2021). However, the abuse of antimicrobial agents, antibiotics, and metal nanomaterials may lead to drug resistance, making the drugs cytotoxic and limiting their potential applications (Song et al., 2020). In addition to finding new antimicrobial drugs as an alternative, many researchers have chosen incorporating antibacterial peptides, such as Epsilon-Poly-L-lysine antimicrobial peptides, in the hydrogel dressings or microneedles to achieve deep bactericidal effects (Wang C. et al., 2019; Xu M. et al., 2019). Brushing cationic polyelectrolyte poly (dially ldimethyl ammonium chloride) onto bacterial cellulose *via* surface-initiated atom transfer radical polymerization could impart a sustained antimicrobial activity to the resultant hydrogels (Yang Z. et al., 2021). Additionally, hydrogels loaded with cationic carbon dots also showed an antimicrobial function (Cui et al., 2021). The prepared cationic carbon dots had an ultra-high positive charge and a small particle size, which could rapidly bind to bacteria and disrupt their surface charge, leading to the apoptosis of bacteria. To prevent the drug resistance of bacteria, incorporating stimuli-responsive substances into hydrogels and combining them with exogenous physical stimuli can achieve effective antibacterial properties. For instance, a catechol-Fe cross-linked matrix with good photothermal conversion ability was combined with quaternized chitosan, and the resultant hydrogel showed good antibacterial properties under NIR irradiation (Liang et al., 2021).

The wound healing process is also severely affected by the failure of macrophage response, during which persistently high levels of pro-inflammatory chemokines and large amounts of ROS are presented at the wound site (Kharaziha et al., 2021). The main idea of preparing anti-inflammatory hydrogels is to give them the ability to promote macrophage polarization, eliminate ROS, reduce pro-inflammatory chemokines, and improve inflammatory inhibitory factors. The most common solution is to add anti-inflammatory drugs to the hydrogels. For example, nanomicelles loaded with pH-responsive nimesulide-loaded anti-inflammatory drugs can promote the transformation of macrophages from M1-type to M2-type, realizing anti-inflammatory function (Wangetal., 2021). In addition to the delivery of anti-inflammatory drugs, loading stem cells and/or genes in hydrogels is another feasible strategy to promote the healing of chronic inflammatory wounds (da Silva et al., 2017). For example, an adhesion GelMA hydrogel containing hyaluronic acid nanoparticles-encapsulated with miR-223 5P mimics was used for chronic wound healing to control the polarization of tissue macrophages (Saleh et al., 2019). Some specific types of ions or elements can regulate the inflammation response. In one study, selenium nanoparticles were loaded into bacterial cellulose/gelatin hydrogel, which could significantly reduce the expression of TNF-α and IL-6, reducing the inflammatory effect during wound healing and promoting wound healing (Mao et al., 2021). Tu et al. (2021) grafted dopamine onto graphene oxide and then loaded the composite into a polydopamine-modified graphene oxide hydrogel. As the catechol on dopamine turned into quinone to trap free radicals, graphene oxide promoted the macrophage polarization, thus conferring the hydrogel with capabilities of anti-inflammatory and capturing ROS. Low levels of reactive oxygen species can promote wound healing, while excessive ROS can hinder wound healing (Xu et al., 2020). Especially in chronic wounds, a sustained inflammation can lead to a high level of ROS, thereby preventing the transition to the next regeneration stage. Therefore, it is particularly important to maintain the balance of ROS level at the wound. Delivering drugs that can scavenge ROS is the most common strategy. For example, thioketone-modified chitosan hydrogel loaded with curcumin could remove excessive ROS to promote wound healing (Yang C. et al., 2021).

### Tissue Proliferation and Remodeling

The proliferation and remodeling periods are the most critical periods of skin wound healing, which mainly involve the formation of granulation tissue, re-epithelialization, angiogenesis, and other events. In the stage of re-tissue regeneration, loading growth factors into hydrogel is an effective manner to improve the wound healing rate. Wu et al. (2016) loaded a-FGF and b-FGF into the thermosensitive heparin-Poloxamer hydrogel, significantly promoting the granulation tissue formation, re-epithelialization, and cell proliferation. However, due to the short half-life and poor stability of growth factors, it is imperative to explore new wound healing agents as an alternative for promoting wound healing. Wang et al. (2021a) isolated a short peptide (RL-QN15) from Rana limnocharis skin secretions, which was proved to promote wound healing in mice. This short peptide could activate the signaling pathway and selectively regulate the secretion of cytokines by macrophages, thereby accelerating the formation of granulation tissue and the regeneration of skin wound.

Hypoxia caused by vascular damage can inhibit wound healing. Therefore, it is extremely important to endow hydrogel trauma excipients with a particular ability to transport oxygen and promote angiogenesis. For example, MnO_2_ nanosheets were loaded into nanoenzyme-reinforced injectable hyaluronic acid hydrogel to provide oxygen to the wound site because the reaction between the high concentrations of reactive oxygen with the MnO_2_ nanosheets could generate oxygen (Wang et al., 2020). In addition to the above endogenous oxygen generation methods, calcium peroxide and catalase were added to GelMA hydrogel to produce oxygen continuously (Erdem et al., 2020). This method could not only improve the cell survival rate loaded in GelMA hydrogel, but also provide oxygen for the wound to promote wound healing.

Angiogenesis at skin wounds plays a key role in the transportation of oxygen and nutrients. Therefore, loading growth factors or peptides that promote angiogenesis in hydrogels has become a feasible strategy. For example, Siebert et al. (2021) developed a 3D printing hydrogel patch coated with VEGF and modified by tetrapod zinc oxide (t-ZnO) particles with photoactivity and antibacterial effect. The controlled release of VEGF could be achieved by chemical modification of t-ZnO and activation with ultraviolet/visible light. Endothelial growth factor modified t-ZnO hydrogel patch showed lower cytotoxicity and improved angiogenesis with an excellent antibacterial capability. In addition to the direct release of angiogenesis-related exogenous growth factors, it is another viable means with the use of growth factors secreted from mesenchymal stem cells or stem cell exosomes by paracrine effect to promote angiogenesis. Eke et al. (2017) loaded adipose-derived mesenchymal stem cells into hydrogel made of a mixture of GelMA and methacrylic acid hyaluronan. The *in vivo* results showed that the hydrogel loaded with stem cells could increase vascularization by three times relative to the case without stem cells, thereby accelerating angiogenesis in the damaged skin. Although the above strategies can accelerate the angiogenesis in the wound defect, sometimes the angiogenesis can be extremely slow or blocked under the influence of uncertain factors. *In vitro* pre-vascularization can be a feasible method. For example, Lei et al. (2019) prepared a hierarchical microchannel network using 3D printing technology, which could reasonably simulate the natural vascular network and showed good structural characteristics.

Scar formation often occurs late in wound healing due to excessive deposition and dislocation of ECM, increased cell density, and chronic inflammation (Xu T. et al., 2019). Scar formation often leads to several complications that can seriously endanger the health of patients. Current research shows that transforming growth factor-β (TGF-β) signal transduction disorder is closely associated with pathological scar formation (Song et al., 2019). Therefore, TGF-β related pathways have been considered as a very promising target for the treatment of abnormal skin scar formation. Yet, TGF-β also plays a very important role in the process of wound healing, including regulating the proliferation and differentiation of epidermal cells and dermal cells and regulating tissue regeneration (Mokoena et al., 2018). Therefore, rational delivery of TGF-β inhibitors at the late stage of wound healing by certain methods becomes a feasible strategy to inhibit scar formation. For example, Zhang J. et al. (2021) loaded TGF-β inhibitor-containing biodegradable microcapsules into a light-cured hydrogel. TGF-β inhibitor was released from the microcapsules by pulse during the late wound healing, resulting in the acceleration of skin wound healing and the inhibition of collagen deposition in wounds and thereby reducing scar formation during wound healing. In addition to TGF-β inhibitors, Shen et al. (2021) designed and prepared a double-layer sodium alginate/polyethylene glycol diacrylate hydrogel for the continuous release of extracellular vesicles at different wound healing stages to achieve a rapid wound healing. In the late stage of wound healing, the small extracellular vesicles secreted by miR-29b-3p-enriched bone marrow-derived mesenchymal stem cells were released from the upper layer of the hydrogel, inhibiting the excessive capillary proliferation and collagen deposition.

Furthermore, external physical stimuli such as mechanical force (Saiding et al., 2020), photothermal (Zhang X. et al., 2021), electrical filed (Korupalli et al., 2021), ultrasound (Lyu et al., 2021), and magnetic field (Shang et al., 2019) at the wound can also accelerate wound healing. Different from the introduction of various drugs or cells into hydrogels to passively promote wound healing, these exogenous signals can directly and actively regulate the behaviors of fibroblasts, keratinocytes, and epithelial cells, modulating the cell behavior to promote wound healing. In one study, the main network of poly (*N*-isopropyl acrylamide) hydrogels were formed by free radical polymerization of *N*-isopropyl acrylamide, a temperature-sensitive monomer, while the semi-crosslinked network was further formed by introducing poly (methacrylic acid) (Hu et al., 2021). The hydrogel could effectively accelerate wound healing in mouse and pig models by promoting angiogenesis, collagen deposition and reducing inflammation through the strong adhesion to tissue and mechanical contraction of the wound. Photothermal therapy is another interesting strategy for promoting wound skin repair. Gao et al. (2019) reported a photothermal hydrogel film, which could convert light energy into heat energy under sunlight irradiation. Excessive local wound temperature can reduce inflammation and promote epithelialization, angiogenesis, and collagen deposition. Mao et al. (2020) prepared a cellulose/MXene composite hydrogel, which could actively regulate the cell behavior and improve the cell viability under the external electric field stimulation, accelerating the wound healing process. Ultrasonic treatment can also accelerate the speed of chronic wound healing. Lyu et al. (2021) fabricated flexible ultrasonic patches, in which ultrasound could accelerate the wound healing by activating RAC1 in the dermis and epidermis, thus effectively treating chronic wounds. Shi et al. (2020) seeded normal human dermal fibroblasts on an anisotropic magnetic hydrogel, and the cells were oriented to grow under the guidance of the magnetic field, indicating that the anisotropic magnetic hydrogel showed a beneficial effect on the skin tissue regeneration.

## Conclusions and Perspectives

Hydrogels have been widely used as a wound dressing for skin wound repair because they are simple to prepare and easy to be modified and functionalized. Hydrogels can be fabricated from many natural and synthetic polymers, meanwhile, they can be loaded with a variety of bioactive substances such as growth factors, proteins, genes, etc., thus making them more biologically active to facilitate wound healing. As a dynamic process, wound healing involves several stages, and each stage involves the synergistic action of multiple factors and cells to promote tissue regeneration. Therefore, modulating the on-demand release of active substances according to the specific needs at the different phases has become a major challenge for current wound dressings. On-demand release must focus on the whole process of wound healing but not only on a particular phase. For example, Ma et al. (2020) reported a multilayer injectable hydrogel, which can sequentially deliver bioactive molecules to meet the requirements for bioactivity and timeline of each wound healing stage. In addition, hydrogel scaffolds with integrated monitoring and therapeutic functions will be a new direction for the development of hydrogel wound dressings in the future. By remotely monitoring wound healing process, a real-time dynamic intervene *in vivo* during the treatment process can be adopted, guiding the targeted therapy. For instance, a wound healing dressing with a double-layer structure can monitor the temperature of the wound in real-time, detect bacterial infection, and release antibiotics through *in situ* UV irradiation to provide an on-demand infection treatment (Pang et al., 2020). Overall, the ultimate goal is to transfer hydrogels from laboratory to industry, from bench to bedside, and finally achieve a true industrialization for the clinical treatment.
